# Tailoring an online breastfeeding course for Southeast Asian paediatric trainees- A qualitative study of user experience from Malaysia and Thailand

**DOI:** 10.1186/s12909-022-03284-z

**Published:** 2022-03-28

**Authors:** Yew Kong Lee, Apichai Wattanapisit, Chirk Jenn Ng, Christopher Chiong Meng Boey, Azanna Ahmad Kamar, Yao Mun Choo, Joyce Soo Synn Hong, Fook Choe Cheah, Swee Fong Tang, Bee Koon Poh, Nalinee Chongviriyaphan, Sirinapa Siwarom, Chonnikant Visuthranukul, Berthold Koletzko

**Affiliations:** 1 Department of Primary Care Medicine, Faculty of Medicine, Universiti Malaya, Kuala Lumpur, Malaysia; 2 School of Medicine, Walailak University, Thasala, Nakhon Si Thammarat, Thailand; 3 SingHealth Polyclinics, Singapore, Singapore; 4 Duke-NUS Medical School, Singapore, Singapore; 5 Department of Paediatrics, Faculty of Medicine, Universiti Malaya, Kuala Lumpur, Malaysia; 6 Universiti Kebangsaan Malaysia Medical Centre, Kuala Lumpur, Malaysia; 7 Department of Paediatrics, Faculty of Medicine, Universiti Kebangsaan Malaysia Medical Centre, Kuala Lumpur, Malaysia; 8 Specialist Children’s Hospital, Universiti Kebangsaan Malaysia Medical Centre, Kuala Lumpur, Malaysia; 9 Department of Nutrition & Dietetics, Faculty of Health Sciences, Universiti Kebangsaaan Malaysia, Kuala Lumpur, Malaysia; 10 Division of Nutrition, Department of Pediatrics, Faculty of Medicine Ramathibodi Hospital, Mahidol University, Bangkok, Thailand; 11 Pediatric Nutrition Research Unit, Division of Nutrition, Department of Pediatrics, Faculty of Medicine, Chulalongkorn University, Bangkok, Thailand; 12 Division of Metabolic Diseases and Nutritional Medicine, Dr. Von Hauner Children’s Hospital, Ludwig-Maximilians-University of Munich, Munich, Germany

**Keywords:** Online learning, Nutrition, Southeast Asia, Malaysia, Thailand

## Abstract

**Background:**

This study explored the user experiences of paediatric postgraduate trainees in Malaysia and Thailand in using a 2 h and 15 min online module for breastfeeding developed for Southeast Asia, which was adapted from existing European online modules for European and German Continuing Medical Education (CME) credits.

**Methods:**

A qualitative study using focus group discussions (FGDs) was conducted with paediatric postgraduate trainees who used an online English-language breastfeeding module in two Thai universities (May 2020, done online) and two Malaysian universities (Sept- Nov 2019, in-person). FGDs explored module usability and utility. Sessions were transcribed verbatim and analysed thematically. The process of coding was done collaboratively by Thai and Malaysian researchers.

**Results:**

Twenty Six resident trainees participated (Thai, *n* = 13; Malaysian, *n *= 13). Ages ranged from 29–34 years old, with 21 females. Nineteen participants had never used online learning modules prior to this. Participants took between 1 to 5 sessions to complete the breastfeeding module. Four themes emerged from their experience. 1) The online learning module was more engaging and detailed than previous lectures, courses and/or books, but lacked hands-on training. 2) Using an online platform facilitated learning as eased navigation and resource searching, however, problems were encountered navigating the module on some devices. 3) Learners preferred less words and more graphics, as this helped them capture key messages. 4) Regionally tailored content elicited a mixed reaction from participants.

**Conclusions:**

Users found that the adapted module compared favourably with previous learning experiences. However, online learning modules lack hands-on training, and implementation should ideally incorporate a mix of both. Consideration of device diversity and preferences for how content was adapted for local settings are needed for tailoring.

**Supplementary Information:**

The online version contains supplementary material available at 10.1186/s12909-022-03284-z.

## Background

e-Learning in medicine has seen tremendous growth and demand. One study estimated that the number of online resources and user access to medical e-learning in Korea has tripled in a single decade [1]. However, online learning material can be challenging for users from low-and-middle income and non-Western countries. A systematic review of medical e-Learning in resource constrained countries highlighted multiple barriers including lack of infrastructure and equipment, lack of face-to-face interaction making engagement difficult, inadequate skilled personnel to support e-Learning, and difficulty in tailoring e-Learning to meet the needs of the local setting, culture and language [2].

Following on from the last barrier mentioned, it is important to develop or adapt material that fits into the healthcare realities and cultural nuances of a country. Most of the studies on adaptation of e-Learning material have focused on Africa and South America, with few studies on Southeast Asia [2]. From 2016 to 2020, German, UK, Romanian, Thai and Malaysian partners worked together to develop a set of e-Learning modules on early nutrition for Thailand and Malaysia as part of the Erasmus + Early Nutrition eAcademy Southeast Asia (ENeA SEA; https://enea-sea.med.lmu.de/) project [3]. A series of four modules (Nutrition & Lifestyle; Breastfeeding; Breast Milk Substitutes; Preterm Nutrition) were adapted from original European modules by Southeast Asian partners to provide localised, practical advice on early nutrition and maternal lifestyle during the first 1000 days of infant development [3]. The adaptation process involved revision of the source material by expert writers and a scientific review board from Malaysia and Thailand, and a process of alpha and beta testing of the modules; the process of development and module features are described in detail elsewhere [3].

This study aimed to explore the online learning experiences of paediatrics postgraduate trainees in Malaysia and Thailand when using an ENeA SEA online module. Exploring the user experiences will allow trainers and educators to understand patterns and barriers to online learning in order to better tailor the implementation of training material for their respective settings.

## Methods

### Design

This qualitative study employed descriptive phenomenology using focus group discussions with postgraduate paediatric trainees in Malaysia and Thailand to explore views and experiences with using an online learning module on breastfeeding.

### Setting

Paediatric postgraduate training in Thailand is a 3-year residency training programme leading up to the Diploma of the Thai Board of Pediatrics certified by the Royal College of Pediatricians of Thailand [4]. Breast milk and breastfeeding is listed as basic medical knowledge in the curriculum [5]. Paediatric postgraduate training in Malaysia is a 4-year postgraduate course leading up to a Masters/Doctor of Paediatrics. In Malaysia, most doctors undergo a two-day breastfeeding course organised by the Ministry of Health Malaysia [6]. Besides national training programmes, both countries have also implemented the World Health Organisation/United Nations Children’s Education Fund (WHO/UNICEF) Baby-Friendly Hospital Initiative and the WHO/UNICEF Infant and Young Child Feeding Counselling Integrated Course to provide training on early infant feeding [7–9].

The original source of the breastfeeding module was a similar module in the Early Nutrition eAcademy (ENeA) project (https://enea.med.lmu.de/), a co-operation between the Early Nutrition Academy and LMU Medical Center Munich [10]. As this was a capacity-building project between EU and Southeast Asian partners, we adapted EU modules as we had access to the source material and EU partners were familiar with this material. In the ENeA breastfeeding module, there are 3 units (Breastfeeding Practice; Benefits; Helping Women Breastfeed); the whole module is accredited for 6 European CME credits or 9 German CME credits.

The ENeA SEA platform is a moodle-based online learning platform with a curriculum written by Thai and Malaysian paediatric and nutrition lecturers; the modules underwent instructional design revision and alpha and beta user testing before the modules were launched. More specifically for this study, the breastfeeding module was revised by a Malaysian university and the writers’ estimated time to complete the module was 2 h and 15 min. The same three ENeA unit titles were retained. Besides adapting the content for regional learners, more videos, interactive learning activities and self-assessment questions were added to the module.

### Participants

Participant inclusion criteria was that they had to be a postgraduate trainee in paediatrics in any of the participating Southeast Asian partner universities in the ENeA SEA project: Chulalongkorn University (CU), Mahidol University (MU), Universiti Kebangsaan Malaysia (UKM), Universiti Malaya (UM). We focused on postgraduate trainees as the original source material was intended for post-degree continuous medical education.

### Recruitment

Postgraduate trainees were invited by research team members (academic lecturers) to participate in the project. If they agreed, a date for the focus group was decided and participants were given a link and instructions on how to access the ENeA SEA Breastfeeding Module about one week before the focus group.

### Data collection

Four focus groups were conducted with each focus group comprised of only respondents from a single institution. In Malaysia, focus groups were conducted face to face in September (UM) and December 2019 (UKM). In Thailand, both focus groups in Thailand were conducted online due to the COVID-19 pandemic (May 2020).

Focus groups were facilitated by two trained researchers and guided by a topic guide that explored the learners’ background, usability, utility and suggestions for the module (Table 1) based on the domains of perceived usefulness and perceived ease of use in Davis’ Technology Acceptance Model [11]. We conducted the Malaysian focus groups in English while the Thai language was used for the Thailand focus groups. The two researchers attempted to probe any different viewpoints from participants in each FGD. Saturation was achieved when no new data emerged from the participants.

**Table 1:** Topic guide for focus group discussion

**Learner background**
1)Could you tell us about your experience in using the online breastfeeding module? 2)What is your general view about this breastfeeding module?
**Usability**
3)Was it easy to go through the module? Probe: Learning platform/ website, self-assessment questions, graphics, interactive elements, page navigation
**Utility**
4)Do you find it useful to learn about breastfeeding early nutrition? Probe for utility: Which sections do you find it helpful? Which sections not helpful? 5)Are the contents (e.g. examples used) relevant to you? Why or why not? Probe: Amount of information, level of information, relevance to local practice
**Improvement/Suggestions**
6)How can we improve it?

### Data analysis

All focus group discussions were recorded and transcribed verbatim into either Thai (FGDs conducted in Thailand) or English (FGDs conducted in Malaysia). The transcripts were typed as text files in Microsoft Word (Microsoft, Redmond, WA, USA). A deductive thematic approach was used to analyse the data [12, 13], and a qualitative data analysis software, NVivo 10 (QSR International, Victoria, Australia), was used for data management. One researcher from Malaysia and one researcher from Thailand coded one transcript respectively from Malaysia and Thailand, to produce an initial coding framework, describing emergent themes in English. The frameworks were compared and iteratively modified via an online meeting. The modified coding framework was used to code the rest of the transcripts and the framework was presented to the larger research team for discussion. Thai quotations were translated into English by a Thai researcher fluent in both languages. The final themes and quotes were discussed by the researchers to ensure the rigor in data analysis.

## Results

A total of 26 participants in four focus groups participated in the study (UM (*n* = 5), UKM (*n* = 8), CU (*n* = 6) and MU (*n* = 7)). Their demographic data is available in Table 2. There are more female than male participants; this is a reflection of the general trend in both Thailand and Malaysia with more female paediatric trainees.

**Table 2:** Participant demographics

Demographic	Overall (*n* = 26)	Thailand (*n* = 13)	Malaysia (*n* = 13)
**Age (range) (*n* = 24)**	29–34 years	29–32 years	30–34 years
**Nationality**			
Thai	13	13	-
Malaysian	13	-	13
**Gender**			
Female	21	12	9
Male	5	1	4
**Ethnicity**			
Thai	13	13	-
Malay	9	-	9
Chinese	2	-	2
Indian	2	-	2
**Ever used online learning before this (*n* = 24)**		(*n* = 11)	
Yes	5	1	4
No	19	10	9
**Device used to access module (*n* = 24)**		(*n* = 11)	
Tablet	10	10	-
Laptop	8	1	7
Desktop and smartphone	2	-	2
Desktop	2	-	2
Laptop and smartphone	1	-	1
Smartphone	1	-	1
**Time taken to complete breastfeeding module (*n* = 23)**		(*n* = 11)	(*n* = 12)
< 2 h	3	0	3
2 to < 3 h	10	4	6
3 to < 4 h	7	6	1
> 4 h	3	1	2
**Sessions to complete breastfeeding module (*n* = 23)**		(*n* = 11)	(*n* = 12)
1	5	2	3
2	8	2	6
3	6	4	2
4	2	2	-
5	2	1	1

Four themes emerged from the focus group sessions: 1) Learners compared online learning with previous learning experiences, 2) Learners found that using an online platform facilitated their learning, 3) Learners preferred less words and more graphics, and 4) Regionally tailored content elicited a mixed reaction.

### Learners compared online learning with previous learning experiences

After using the online material, participants intuitively compared this to their previous learning experiences. This was done in three ways.

First was a comparison with existing training courses on the same topic. This was a common theme for the Malaysian participants who mentioned the twenty-hour breastfeeding course that doctors are required to complete during their houseman training. They mentioned that the online module was better as it was clearly structured and allowed them to pace their learning. Compared to the twenty-hour face-to-face lectures, topics were similar, but included more detailed higher-level content. However, they noted the online module was missing practical elements, such as going to wards to conduct hands-on massage and talking to patients.

*We went through the twenty hours breastfeeding course previously. Frankly to say it’s a bit dry. Twenty hours and then you just sit there and listen to the talk while doing this e-module is actually at our own time. (M5, 31 y.o. male, Malaysian)*

*It’s (the online module) detailed, the contents. Comparing to the courses that we have gone to- the twenty hours breastfeeding course- is for like all staff, including also the non-doctor staff, so they make it more for a layman (M2, 34 y.o. female, Malaysian)*

*Like massaging (breast massage) part. Went to all the wards and do that. That was really helpful and is a hands-on session. We can ask from both sides like interactive, but from the online courses is not much of the hands-on (M6, 32 y.o. female, Malaysian)*

The second way it was compared was by contrasting online learning with face-to-face learning. Thai participants mentioned that there were advantages to having face-to-face lectures compared to online learning, such as getting distracted easily or losing motivation when no lecturer was present. Thus, one suggestion was to include more recorded lectures in the online course.

*I personally prefer face-to-face learning. If I read (the content) on my iPad, it is easy to be distracted. It is easier to remember (the content) by listening to someone. This is my personal learning style. The e-Learning requires lots of concentration and attention. It is not my type. (T7, 30 y.o. female, Thai)*

*The advantage is I can study at any time and any place. I do not need to finish the whole module at one time; I can come back later and continue the lesson. However, the drawback is the attention can be distracted by external factors (laughing). It is different from face-to-face learning (T4, 30 y.o. female, Thai)*

*It is not different from reading a book. There are many texts. I expected to see several video clips, but the video was not rich in terms of its content. It is an introductory video. The rest consists of text only. It is not much different from reading lecture notes. The module just separates the contents into small topics. I expect to see more video clips that emphasise the important points. Videos that contain a recorded lecture are feasible to be on the online module. The videos should be concise. It is impossible to see a 4-hour video. A 1 hour-video combine with self-study can be useful. I can read more contents that I am interested in. It will probably help me to focus on the contents. (T5, female, Thai)*

The third comparison was made with books. This was done by Thai participants. One Thai participant mentioned that the experience in using the online material was “not any different from reading a book” (T5, female, Thai). Another felt that compared to books, online learning was better as the material could be navigated in smaller segments compared to longer book chapters. One last point was related to the interactivity of online material; one Malaysian mentioned that e-Learning was more fun compared to reading a booklet, while a Thai participant said that compared to a book, learners could actively test their learning effectiveness through the interactive activities.

*Compared between the e-Learning and reading books, both ways require huge reading efforts. Anyway, the e-Learning is more attractive because there are videos, diagrams, and pictures. Moreover, the chapter summary helps me understand. I can know by doing quizzes whether my understanding is correct or incorrect. Compared to conventional lectures, it is different because lecturers organise the lesson and can offer two-way communication. To cover all the contents, lectures may take a long time. The e-Learning can be separated into smaller sections that we can attend several sessions. The whole thing in one session can induce ‘brain swelling’. (T10, 30 y.o. female, Thai)*

*The e-Learning is more effective than reading books. Although there is no face-to-face participation, the e-Learning is quite interactive. If I get a 30-page lecture note, I may not have the motivation to read it. The e-Learning may take as long as reading the lecture note. Otherwise, I give up reading. The advantage of the e-Learning is offering interactive learning. The videos are interesting and can be the feedback material to recheck my understanding. Compared to lectures, I am not sure which one (e-Learning or lecture) is better. If I have any questions, I could ask the lecturer directly and I would receive the response immediately. I cannot decide whether the e-Learning is better than lectures, but it is better than reading books or lecture notes. (T12, 30 y.o. female, Thai)*

To make up for perceived deficiencies in the portal, some participants suggested a hybrid combination of including either a hands-on practical (for Malaysians), or face-to-face lecture (for Thais) after the module. Technology could be used to integrate the two platforms such as embedding a QR code for the online modules in a lecture.

*Can I get both? I mean a combination of a face-to-face lecture and the e-Learning. People who cannot attend the lecture can go to the link or QR code to the e-Learning. The advantage of the e-Learning is its convenience. Participants can learn at any time and any place. Its disadvantage is distraction. Excessive texts are boring. I have to break and come back. Listening a lecture has a good thing that I can interact with the lecturer. Sometimes, I cannot understand by self-reading. Lecturers can help. The limitation (of face-to-face lectures) is that not all people are available for a lecture. (T1, 30 y.o. female, Thai)*

*Maybe (the training) can be done in two parts. First, the self-learning like this. And then must complete other part, which is hands on, maybe can do later. (M3, 30 y.o. female, Malaysian)*

### Learners found that using an online platform facilitated their learning

Users mentioned ways in which the online platform helped them learn. One theme was that learning was more convenient, as they could access material instantly anytime and anywhere, were able to access and break the module up into multiple sessions and could easily navigate back and forth between the module content.

*E-module is easy for me. I guess the best part is I can stop it halfway, do other stuff, then go back to it. That is the flexibility in there. And one thing is we can go back and revisit the same thing…I mean the information is already there on it. Like if you're going for a physical course sometimes like reading materials are like not provided or you like take your own. This one you know where you want to find the information. You know where it is so easier to revisit. I think that is the most important part. (M3, 30 y.o. female, Malaysian)*

*I can eat, drink when listening or reading so I think it's better in a way that it’s in my own time compared to the previous twenty hours breastfeeding course. (M5, 31 y.o. male, Malaysian)*

Being online already was also advantageous for learning as they supplemented the module with other online material. As they were already online when using the module, participants mentioned that they would head elsewhere online to search for more information. This could be for two reasons, either to check if they had understood the module correctly, or to search for additional, further information.

*Sometimes, it (problem) is the language. Also, the latter part (of the module) is about organisation management and so on. I searched on the internet to find out. (T10, 30 y.o. female, Thai)*

*Meaning that you can search in Google or what does this mean, what does, what, what do this mean and then if you want to search for further references, then you can search as well. Do your own readings as well…because it’s online. (M2, 34 y.o. female, Malaysian)*

However, using an online platform presented some technical challenges. Users mentioned issues with webpage navigability and usability due to device functions. For webpage navigability, the use of tablets was problematic for some (Thai users were using tablets) as buttons on the tablet browser did not display correctly and they accidentally clicked the wrong navigation button. Handphone users in Malaysia reported issues with truncated text and difficulties with drag-and-drop activities.

*Some pages were disappeared. For example, I clicked ‘next page’, it moved to the final page. Many pages were skipped. I did not read them. (T4, 30 y.o. female, Thai)*

*It did not happen for the quiz, but it occurred in the main text. As my friend said, I initially studied, then clicked a button, optional further information, or something. It was close to the next page button. Ah, I accidentally chose the next page. I realized that I should click the optional further information button and read the contents inside. I had to go back - that is to say. (T1, 30 y.o. female, Thai)*

*Another difficulty was because I accessed using handphone. So the (quiz) question, one of the question is too long so it cannot appear. (M1, 31 y.o. female, Malaysian)*

*I think I've experienced whereby the one of the questions where you have to drag the answers and go into the box. So when I use the handphone, it cannot really drag upwards. It won't automatically scroll down. So you cannot put it in the answer box. (M2, 34 y.o. female, Malaysian)*

### Learners preferred less words and more graphics

There were interactive and design elements in the online module that participants commented on. A number of participants from both Malaysia and Thailand mentioned that the presentation was too wordy or lengthy. To alleviate this, they suggested more tables or diagrams.

*For me I think the content is very good just that is a bit lengthy for me. If I read one shot feel like very lot of things to absorb. Maybe they can put in table or diagram. (M4, 30 y.o. female, Malaysian)*

*The content is quite a lot. For healthcare providers, it is a big task to learn. The content is useful for doctors, nurses, or public health professionals, but there are too many texts. Information is too much. (T2, 30 y.o. female, Thai)*

*I agree with others. It is different from our ordinary breastfeeding lessons. The module covers every aspect, from milk production to recommended diet. However, I feel this module is too wordy. I skip some contents because the texts are too many. It should be more interesting. (T5, female, Thai)*

Thai participants mentioned that the graphics and videos made by the Malaysian team was well designed, and that the use of graphics helped to improve understanding of key messages.

*It is nice-looking. I mean the pictures are clear. The diagrams are beautiful and easily understandable. (D12, 30 y.o. female, Thai)*

*I personally like figures, diagrams, and tables. They are helpful and concise. (T9, 31 y.o. female, Thai)*

Two elements were highlighted as helping them understand the focus or key points of the module. These were the interactive quizzes, and also “Key message” summary boxes at the end of each unit.

*The content is good and concise. At the end of each section, the summary helps understand the key points. I like the test that I can check my knowledge after the study. (D12, 30 y.o. female, Thai)*

### Regionally tailored content elicited a mixed reaction

When talking about some of the content that had been tailored for Malaysia and Thailand (e.g. local practices such as post-pregnancy confinement, comparison between haematinic micronutrients in Malaysia and Thailand), participants had mixed reactions. Some took an inter-regional perspective and found it interesting to compare between country practices and recommendations to see how different contexts affected practice guidelines. Some took a more critical perspective; they either did not see the relevance of learning about practices from other countries or they questioned why there was so little data on the Southeast Asian region.

*I think the content about the examples of international policies, the length of maternity leave and salary during the leave, is not useful for me. (T6, male, Thai)*

*I like the e-module because it is a collaboration for Malaysia and Thailand so from the information and knowledge provided I can compare all the requirements. The knowledge is given based on the countries, so I'm not just focusing on my country. I can compare with others…. for example, for the dietary or micronutrients that they recommends is not all fixed. It’s different between all the countries. I think it’s good ‘cause for example, the haematinics from the section on micronutrients there is table comparing Malaysia and Thailand. We are using the haematinics so I can see that Thailand is using something else instead of the haematinics that we have like iron, folate, Vitamin B12 and the other one I don’t know. But they didn’t include all the micronutrients that we did. They are using other things depending on their state of health (M3, 30 y.o. female, Malaysian)*

*But just one thing is very funny because we are in South East Asia but I find that a lot of information there is no data for South East Asia. It's lack of data so I find that is a bit funny ‘cause if you want us to participate, you should have at least some data concerning the South East Asian area. (M8, 33 y.o. male, Malaysian)*

Another regional issue was language proficiency. As the modules were only available in English, some users commented that limited English proficiency increased the time required and taxed their ability to focus on the nutritional aspect of the course.

*It takes time because of English language. I have to reread several times. Especially, for the tests, I have to pay more attention on the question. I think I use more time. If this content is converted into our language, I can complete within a shorter period. (T7, 30 y.o. female, Thai)*

*The test questions are complicated such as every item is correct, with one ‘except’. I feel like I am doing an English proficiency test rather than the test of knowledge of nutrition. (T11, 29 y.o. female, Thai)*

## Discussion

This study aimed to compare the experience of postgraduate paediatric trainees in using an online module between Thai and Malaysian learners. Some general patterns were that the majority of Thai users accessed the platform through tablets, while Malaysians used mostly laptops. Possible reasons for high tablet usage in Thailand could be high internet usage contributing to Thai user preferences for internet friendly devices such as smartphones and tablets [14], and a pattern of increased use of tablet computers by residents compared to undergraduate medical students [15]. Most of the participants (19/26) said that they had never used online learning modules before even though the Thai focus group meetings were conducted in May 2020 after the first COVID-19 case in Thailand was reported in January 2020 [16]. Also, only Thai users mentioned English as an issue, while Malaysians did not; Malaysia is a former British colony and has high English proficiency [17]. Although not statistically representative, the results suggest that Thai participants had longer completion times and took more sessions to complete the module; this may be due to language barriers. To assist in language issues, we have since made Thai [18] and Malay [19] translations available.

In our study, learners compared their online learning experience with existing learning methods such as face to face lectures, training courses and books. In general, they were positive about learning online as they could break up the lesson and pace their learning in a flexible manner. While this advantage is to be expected, the fact that it was mentioned by quite a few participants shows that this form of asynchronous learning with online platforms is not yet widespread in lower-middle income countries. Without available online learning resources, audio-visual lectures continue as the primary means of medical teaching; this is done either synchronously via video conferencing, or asynchronously via pre-recorded slideshows [20]. Issues with this approach include screen fatigue and distraction for students and lecturers [20]. Consequently, surveys from LMICs during the COVID-19 period show that students prefer traditional teaching compared to online teaching [21], and that comprehension from face-to-face lectures is better than online teaching [22]. This shows that investment into developing online learning material is important in order to improve student acceptability. Faculty development programmes should include training on systematic approaches to e-Learning development and implementation (such as the ADDIE framework) and instructional design approaches to achieve clinical learning outcomes online [23]. The students’ preferences for media-rich material were possible as the ENeA SEA project had a Distance Learning Expert Board to develop and implement media; faculties should consider investing resources into e-Learning teams (including e-Learning researchers, media developers, learning technologists, and software developers) [3].

Learners in our study highlighted that being online was helpful as they could easily search for additional or clarifying information. This self-initiated search for material outside of the provided syllabus is supported by the self-directed learning paradigm for adult learning [24] and studies describing postgraduate medical students as strategic learners who actively move between deep and surface learning when it is required [25]. Following on from the recommendation that adult learning theory needs to pay attention to the contexts in which learning occurs [26], our study suggests that developers of postgraduate medical education can leverage the online learning environment by including links to online resources, using open-access articles as much as possible, and ensuring that online platforms work well on the preferred devices in the target region.

This study shed light on how students in Southeast Asia responded to material that had been adopted by a multinational team and adapted from a European setting to suit their local context. This adopt-and-adapt approach fits in between the spectrum of fully imported material (which may not fit with local nuances) and material written from scratch (which is time-consuming and may lack the relevant content writers). In Malaysia and Thailand, the process of cultural adaptation is often applied only to patient material or programmes where emphasis is on being sensitive to cultural values such as religious beliefs or taboos [27–29]. Similarly, in our study, local cultural practices related to breastfeeding (such as confinement) were included. Although participants in this study appreciated this local flavour, it is likely that they are already familiar with these local beliefs. Some participants showed keen interest in comparisons of practice variations such as differing paediatric nutrient guidelines between Malaysia and Thailand. Whether an etic approach (comparing between or across cultures/practices) or an emic approach (comparing within cultures/practices, with little reference to other settings) [30] is more suitable when adapting for local settings is something that should be explored in further studies, as both views were expressed by our participants.

### Limitations

The results are limited to users’ experience with just a single module out of the six modules and we were unable to obtain their responses to a longer course. Another limitation was that all four universities were in urban areas with good internet coverage (the capital cities of Malaysia and Thailand respectively); we are unable to explore how users with issues such as poor network or limited device access, which are common in developing countries, would otherwise experience online learning. Finally, although the Thai interviews were conducted in the midst of the COVID-19 pandemic, we are unable to determine if this has impacted their experience in using the online learning module.

## Conclusions

Tailoring existing online courses for local health and cultural settings was a feasible and acceptable method of developing a locally relevant course for Southeast Asian settings. Student background (e.g. device preferences), local training (e.g. past courses), and comparative policy and practice (e.g. practice guidelines) affected how users experienced the adapted course; consider these factors when adapting or developing online courses in Southeast Asian settings. Continuous medical education providers in low resource settings should leverage the online environment by adapting existing courses, utilising open access links, and ensuring that online platforms work well on the preferred devices in the target region.

## Supplementary Information

**Additional file 1.** ENeA SEA Project Group.

**Additional file 2.** Focus Group Discussion Topic Guide.

## Data Availability

All data generated or analysed during this study are included in this published article [and its supplementary information files].

## Abbreviations

ENeA SEA: Early Nutrition eAcademy Southeast Asia
ENeA: Early Nutrition eAcademy
CU: Chulalongkorn University
MU: Mahidol University
UKM: Universiti Kebangsaan Malaysia
UM: Universiti Malaya
