# Supplementary material for: Tailoring an online breastfeeding course for Southeast Asian paediatric trainees- A qualitative study of user experience from Malaysia and Thailand
Source: BMC Med Educ. 2022 Mar 28;22:209. doi: 10.1186/s12909-022-03284-z (PMC8960711; doi:10.1186/s12909-022-03284-z)
Supplement: Supplementary file 1 — Additional file 1. ENeA SEA Project Group. [file 12909_2022_3284_MOESM1_ESM.docx]

**ENeA SEA Project Group**

The ENeA SEA project group: Yew Kong Lee^1^, Apichai Wattanapisit^2^, Chirk Jenn Ng^1^, Christopher Chiong Meng Boey^3^, Azanna Ahmad Kamar^3^, Choo Yao Mun^3^, Joyce Soo Synn Hong^4^, Fook Choe Cheah^5^, Swee Fong Tang^6^, Bee Koon Poh^7^, Nalinee Chongviriyaphan^8^, Sirinapa Siwarom^8^, Chonnikant Visuthranukul^9^, Berthold Koletzkon (Project Leader) ^10^, Brigitte Brands (Associate Coordinator) ^10^, Rungrawee Loipimai (Administrative Project Management) ^10^, Shweta Feher (Administrative Project Management) ^10^, Keith M Godfrey ^11^, Sunhea Choi ^11^, Philip Calder ^11^, Sirinuch Chomtho ^9^, Orapa Suteerojntrakool ^9^, Jaraspong Uaariyapanichkul ^9^, Naiyana Nujankaew ^9^, Janya Sukhapan ^9^, Naipapohn Chuenmeechow ^9^, Sungkom Jongpiputvanich ^9^, Umaporn Suthutvoravut ^8^, Chatchay Prempunpong ^8^, Oraporn Dumrongwongsiri ^8^, , Boonpraserd Treerayapiwat ^8^, Sujitraporn Ratanarom ^8^, Lucy Chai See Lum ^5^, Ruey Terng Ng ^5^, Zahiruddin Fitri Abu Hassan ^12^, Azmi Mohd Tamil ^6^, Helmi Norman ^13^, Stefan Trausan-Matu ^14^, Mihai Dascalu ^14^, Gabriel Gutu-Robu ^14^

**Affiliations**

1. Department of Primary Care Medicine, Faculty of Medicine, Universiti Malaya, Malaysia.

2. School of Medicine, Walailak University, Nakhon Si Thammarat, Thailand.

3. Department of Paediatrics, Faculty of Medicine, Universiti Malaya, Malaysia.

4. Universiti Kebangsaan Malaysia Medical Centre, Kuala Lumpur, Malaysia.

5. Department of Paediatrics, Faculty of Medicine, Universiti Kebangsaan Malaysia Medical Centre, Kuala Lumpur, Malaysia.

6. Specialist Children’s Hospital, Universiti Kebangsaan Malaysia Medical Centre, Kuala Lumpur, Malaysia.

7. Department of Nutrition & Dietetics, Faculty of Health Sciences, Universiti Kebangsaaan Malaysia, Kuala Lumpur, Malaysia.

8. Division of Nutrition, Department of Pediatrics, Faculty of Medicine Ramathibodi Hospital, Mahidol University, Bangkok, Thailand.

9. Pediatric Nutrition Research Unit, Division of Nutrition, Department of Pediatrics, Faculty of Medicine, Chulalongkorn University, Bangkok, Thailand.

10. Division of Metabolic Diseases and Nutritional Medicine, Dr. von Hauner Children's Hospital, Ludwig-Maximilians-University of Munich, Germany.

11. MRC Lifecourse Epidemiology Unit and NIHR Southampton Biomedical Research Centre, University of Southampton and University Hospital Southampton NHS Foundation Trust, Southampton, UK.

12. Department of Building Surveying, Faculty of Built Environment, Universiti Malaya, Malaysia.

13. Department of Paediatrics, Faculty of Medicine, Universiti Kebangsaan Malaysia, Bangi, Malaysia.

14. University Politechnica of Bucharest, Bucharest, Romania.
