# Supplementary material for: Tailoring an online breastfeeding course for Southeast Asian paediatric trainees- A qualitative study of user experience from Malaysia and Thailand
Source: BMC Med Educ. 2022 Mar 28;22:209. doi: 10.1186/s12909-022-03284-z (PMC8960711; doi:10.1186/s12909-022-03284-z)
Supplement: Supplementary file 2 — Additional file 2. Focus Group Discussion Topic Guide. [file 12909_2022_3284_MOESM2_ESM.docx]

**Focus Group Discussion Topic Guide for clinical masters trainees**

Objective:

- To explore the experience of trainees in using the EneA SEA online breastfeeding module.

Preamble:

- Ice breaking
- Explain the objectives
- Go through the patient information sheet (PIS) and explain how the focus group discussion will be done
- Explain no right or wrong answers
- It is the opinion that we are interested in
- Feel free to respond to what others are saying
- Explain the need for audio-recording
- Obtain consent for the focus group and audio-recording

Background:

1. Could you tell us about your experience in using the online breastfeeding module?
   1. eLearning experience
2. What is your view about this breastfeeding module?

Usability

1. Was it easy to go through the module (Probe for usability)
   1. Probes: Learning platform/ website, Self-assessment questions, Graphics, interactive elements, Page navigation

Utility

1. Do you find it useful to learn about breastfeeding early nutrition? (Probe for utility)
   1. Which sections do you find it helpful? Which sections not helpful?
2. Are the content (e.g. examples used) relevant to you?
   1. Why or why not? Probes: amount of information, level of information, relevance to local practice

Improvement

1. How can we improve it?

**End**

Thank you for participating in this focus group discussion.
